# First artificial hybrid of the eel species *Anguilla australis *and *Anguilla anguilla*

**DOI:** 10.1186/1471-213X-11-16

**Published:** 2011-03-13

**Authors:** Erik Burgerhout, Sebastiaan A Brittijn, Tagried Kurwie, Paul Decker, Ron P Dirks, Arjan P Palstra, Herman P Spaink, Guido EEJM Van den Thillart

**Affiliations:** 1 ZF-screens BV, Niels Bohrweg 11, 2333 CA Leiden, The Netherlands; 2 Mahurangi Technical Institute, PO Box 414, Warkworth, New Zealand; 3 Leiden University, Institute of Biology Leiden, Gorlaeus Laboratories, POB 9502, 2300RA Leiden, The Netherlands

## Abstract

**Background:**

Studies on artificial hybridization of different *Anguilla *species were conducted recently, i.e. female *A. australis *with male *A. dieffenbachii*, and female *A. japonica *with male *A. anguilla*. The existence of these artificial hybrids was however not demonstrated by independent genetic methods. Two species - *A. anguilla *and *A. australis *- that are phylogenetically close but have different sexual maturation times (12-25 weeks and 6-8 weeks, respectively), were expected to produce favourable hybrids for reproduction studies.

**Results:**

A modification of the protocol for the reproduction of *Anguilla japonica *was used to produce eight-day *Anguilla australis *larvae, with a success rate of 71.4%. Thus ten out of 14 females produced eggs that could be fertilized, and three batches resulted in mass hatching. Hybrid larvae from female *A. australis *x male *A. Anguilla *survived for up to seven days post fertilization (dpf). The early development of the hybrid showed typical characteristics of *A. anguilla *tail pigmentation at 50 hours post fertilization (hpf), indicating expression of genes derived from the father.

**Conclusions:**

In this paper we describe the first production of hybrid larvae from male *A. anguilla *and female *A. australis *and their survival for up to 7 dpf. A species-specific nucleotide difference in the 18 S rDNA gene confirmed that genes from both *A. australis *and *A. anguilla *were present in the hybrids. The developmental stages of the hybrid eel embryos and larvae are described using high resolution images. Video footage also indicated a heart beat in 5-dpf larva.

## Background

A number of research groups have attempted artificial reproduction in various species of eel: *A. japonica *[1-5], *A. anguilla *[[6-9], Tomkiewicz, unpublished data], *A. dieffenbachii *[10], *A. australis *[[10], Kurwie, unpublished data], and *A. rostrata *[10]. Some Japanese scientists have also overcome major problems associated with developing artificial feeds for larvae and have successfully produced leptocephalus larvae [11] and glass eels [12,13]. Tanaka and his co-workers used a mix of shark egg powder, soya peptide, minerals, vitamins and krill paste [11] to develop a successful feed for *A. japonica*. Further research is, however, needed to develop suitable diets and rearing techniques for the production of larvae of other *Anguilla *species and their hybrids.

European eel (*A. anguilla*) females have a much slower, and widely-variable, response to hormonal stimulation [9] when compared to females of other freshwater eel species (e.g. *A. japonica *and *A. australis*). At the onset of the natural spawning migration, the gonadosomatic index (GSI) of *A. anguilla *females is close to 2% [A Palstra, unpublished data] and they are still in a previtellogenic state when they migrate to sea. However, females of *A. australis *have a higher GSI, of up to 4% [14], indicating that they are sexually more advanced than *A. anguilla *at the same stage The same holds true for *A. japonica*, which has a GSI of up to 4% at the commencement of its spawning migration [15]. Induction of vitellogenesis and final maturation in *A. australis *requires approximately six to eight weekly hormonal injections [[10], Kurwie, unpublished data] while 9-12 injections [4], or 6-15 weekly injections [11], are required for *A. japonica *and up to 12-25 weekly injections for *A. anguilla *[7-9].

There are several reasons for testing hybridization between European and New Zealand short finned eels. There are large differences in silver eel maturation states between these species. In contrast to the stage reached by *A. australis*, silver eels of *A. anguilla *have not yet commenced vitellogenesis. Shortening the artificial trajectory may overcome vitellogenic abnormalities, resulting in higher gamete quality and higher success rates of fertilization, hatching and larval development.

*Anguilla anguilla *is listed by the IUCN as critically endangered [16], which raises some problems in association with the culture of this species. Farming is reliant on the influx of wild glass eel, thereby pressurizing wild stocks. Breeding for aquaculture is, nevertheless, supposed to take pressure off wild stocks. Therefore, the hybridization of *A. anguilla *with a species such as *A. australis*, that has a short artificial trajectory, may be a suitable option for aquaculture. Since maturation levels at silvering are very different in the parent species, it is quite possible that the maturation level of the hybrid at the silver stage would be far more advanced than that of the European silver eel. Furthermore, since *A. australis *lives in the southern hemisphere, its migration is in January-June [17], in contrast to *A. anguilla*, which migrates in October-November. To gain insights into the combination of the properties of *A. australis *and *A. anguilla *present in hybrids, it is useful to study eel reproduction and to compare the early ontogeny of these species including their hybrids.

A number of studies on hybridization of various eel species have been previously conducted: for example, female *A. australis *with male *A. dieffenbachii *[10], and female *A. japonica *with male *A. anguilla *[18]. The existence of the artificial hybrids has not, however, been demonstrated by independent genetic methods. In contrast, genetic evidence for natural hybrids between the Atlantic species *A. rostrata *and *A. anguilla *has been demonstrated [19]. Since *A. anguilla *and *A. australis *are phylogenetically more closely related than some other hybrids (for example, *A. anguilla *and *A. japonica *[20]), we hypothesized that hybridization between the former two species would be possible. In this paper we describe experiments on the hybridization of *A. anguilla *and *A. australis *and post-fertilization survival levels. Investigations into the18 S rDNA gene -- for the purpose of genetic validation -- are also described.

## Results

### Reproduction

After 4 weeks of injections with hCG, the first *A. australis *male started to spermiate and all males had spermiated within 6 weeks. After 9 weeks the injections were stopped. Three of the 15 *A. anguilla *males produced sperm after 5 weekly injections and all males of *A. anguilla *had produced sperm after 6 weekly injections. Before use, the males received a booster hCG injection to reactivate spermiation.

In most females, hormone treatment resulted in a rapid increase in body weight after 9-13 injections with salmon pituitary extract (SPE). Ten of the 14 females (71.4%) ovulated once and seven females ovulated twice during this study. The second ovulation was induced 2 weeks after the first ovulation by a single injection of 20 mg SPE/kg dissolved in 1 mL 0.9% saline, one priming injection of SPE, and an injection with 17, 20β-dihydroxy-4-pregnen-3-one (DHP) one day later. Three females did not respond to the SPE treatment and one female died after the DHP injection, just before ovulation. In total, three out of the ten batches of eggs produced larvae (33.3%). The larvae of two of the three batches from *A. australis *x *A. australis *stayed alive for 5 dpf, and the larvae from one batch of the hybrid *A. australis *x *A. anguilla *stayed alive for 7 dpf. Some of the eggs from one batch of *A. australis *were reared at 25°C, which resulted in larvae that survived until 8 dpf.

### Embryogenesis and early larval development of *A. australis*

**Figure 1:**
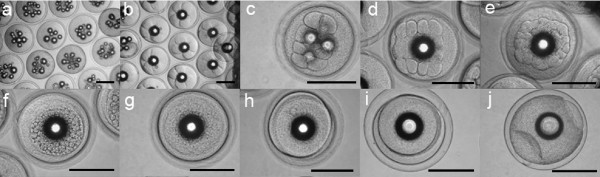
**Embryogenesis of *A. australis***. Early ontogeny until the late gastrula stage: a) Fertilized eggs, ~0.5 hours post fertilization (hpf); b) 4-cell stage, ~1.5 hpf; c) 8-cell stage, ~2 hpf; d) 16-cell stage, ~2.5 hpf; e) 32-cell stage, ~4 hpf; f) morula stage, ~6 hpf; g) blastula stage, ~7 hpf; h) and i) early gastrula stage (shield stage), 9-13 hpf; j) late gastrula stage, 20 hpf. (Scale bar = 1 mm).

**Figure 2:**
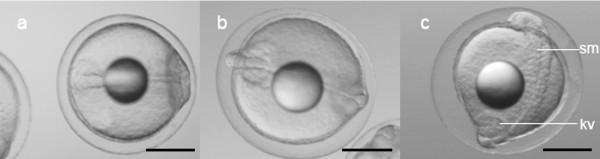
**Somitogenesis in *A. australis *embryos**. Stages of somitogenesis: a) 6-7 somites, ~24 hpf; b) 11 somites, ~26 hpf; c) 17 somites, ~30 hpf. (Scale bar = 0.5 mm; kv = Kuppfer's vesicle; sm = somites) Note: due to the limited depth of field of the microscope in relation to the size of the embryos, not all structures can be shown simultaneously.

After fertilization (Figure 1a), developing eggs floated to a level just below the surface of the water, resulting in a clear separation from undeveloped eggs, which sank. Cell divisions occurred every 30-60 minutes. The 4-cell and 8-cell states were observed at the 1.5 hpf stage (Figure 1b) and the 2 hpf stage (Figure 1c), respectively. The 16-cell stage and 32-cell stage were observed at 2.5 hpf (Figure 1d) and 4 hpf (Figure 1e), respectively. The morula stage (Figure 1f) and the blastula stage (Figure 1g) were observed at 6 hpf and 7 hpf, respectively. An embryonic shield started to form between 9 and 13 hpf (Figure 1h and 1i), and the late gastrula stage was observed at about 20 hpf (Figure 1j). Somitogenesis started between 20 and 24 hpf (Figure 2). The Kuppfer's vesicle (Figure 2c, for description see [21]) was observed at about 26 hpf, and the first heartbeat occurred at about 43 hpf.

**Figure 3:**
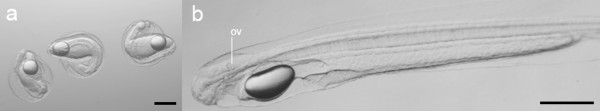
**Hatching of *A. australis***. Structures in larvae: a) hatching larva, ~45 hpf; b) larva at 2.5 dpf. (Scale = 0.5 mm; ov = otic vesicle with otoliths).

Neutral buoyancy of the eggs was observed just before hatching. At approximately 43 hpf the embryos started to hatch and at that time had approximately 40 somites (Figure 3a; 45 hpf). After hatching (Figure 3b; 2.5 dpf) the larvae (at first C-shaped) were positioned upright in the water column, probably due to the position of the oil droplet. It appeared that the larvae were neutrally buoyant at 35 ppt. The larvae were immobile, except when disturbed by light or vibrations, which caused very fast and short horizontal movements. During sampling procedures (by pipette) the larvae avoided suction, and swam in the opposite direction at speeds of up to several body lengths per second. Sampling caused mechanical damage to larvae, followed by death within a short period. Discoloration of the brain and neural tube was observed within a few seconds, followed by cellular breakdown of the larvae.

**Figure 4:**
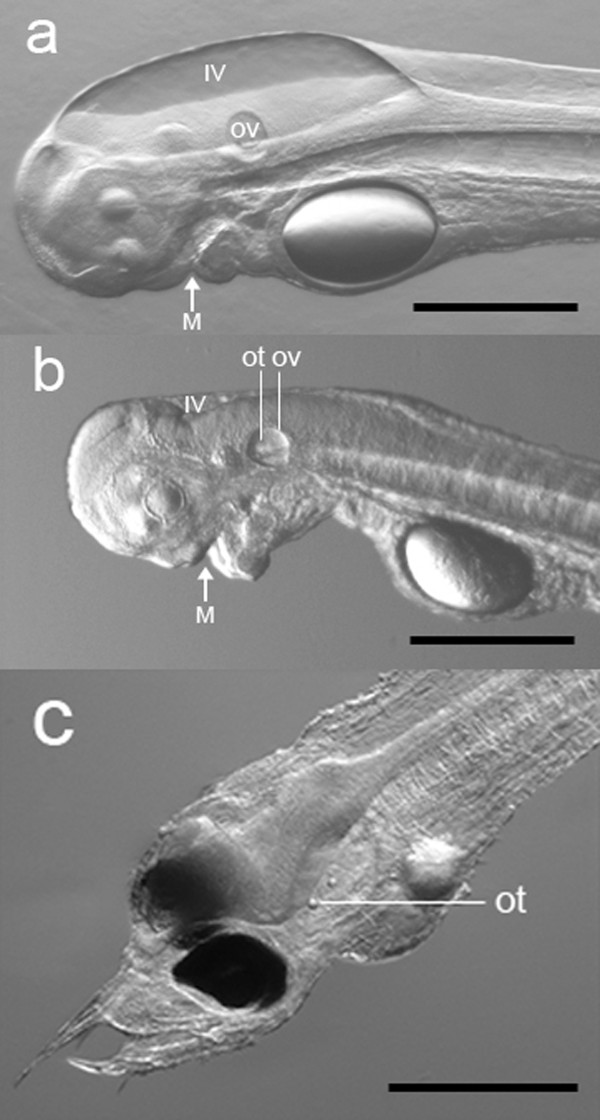
**Head development of *A. australis***. Head structures of *A. australis*: a) 5 dpf larva; b) 6.5 dpf larva; c) 8 dpf larva reared at 25°C (representing a stacking of two illustrations of the same larva). Note the remarkable differences between 5 dpf and 6.5 dpf larvae in the development of the 4^th ^ventricle (IV) (with a decrease in volume) and the protrusion of the mouth (M). Between 6.5 dpf and 8 dpf, the formation of teeth commences, the angle of the head increases, and the eyes become pigmented. (Scale bar = 0.5 mm; ov = otic vesicle with otoliths; ot = otoliths).

**Figure 5:**
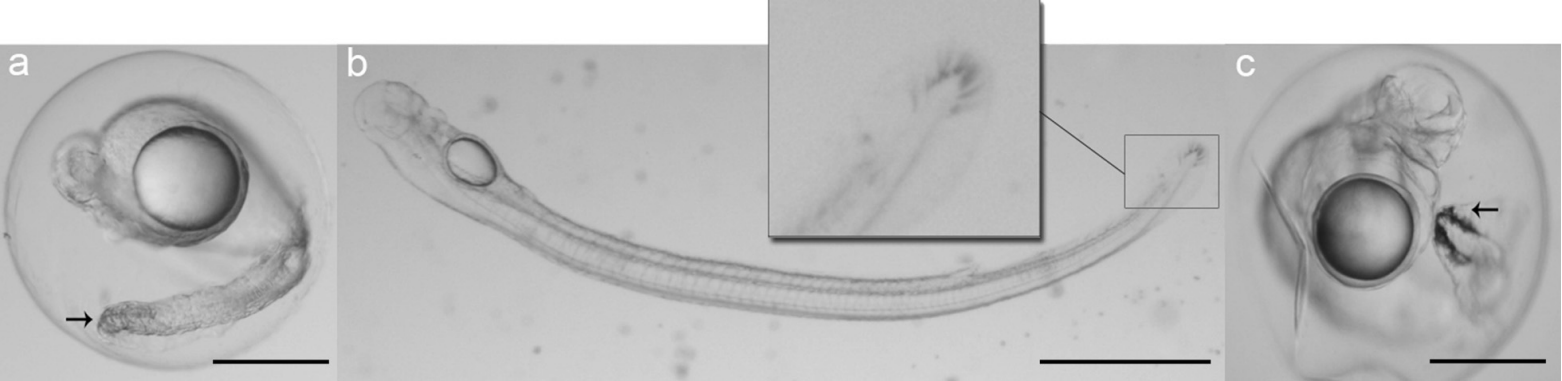
**Timing of tail pigmentation**. Differences in timing of tail pigmentation in *A. australis *and the *A. australis *x *A. Anguilla *hybrid: a) hybrid species at ~50 hpf; b) *A. australis *at 6 dpf; c) *A. anguilla *at 60 hpf. (Scale bar in a,c= 0.5 mm, in b= 1 mm; arrows indicate pigmentation of the tail).

Larvae elongated during development and lateral neuromast cells on the flank were observed at 68 hpf. Head development showed remarkable changes over time, especially a decrease in volume of the 4^th ^ventricle between 5 and 6.5 dpf (Figure 4a, b), the protrusion of the mouth and development of teeth between 5 and 8 dpf, and pigmentation of the eyes at 8 dpf (Figure 4c). The angle of the head also increased in such a way that the mouth protruded anteriorly. At 8 dpf the larvae showed well developed teeth and a straightened head, indicating that they had reached the feeding stage. No visual differences in development between larvae from *A. australis *and the hybrid species were observed, except for the development of tail pigment cells, which were already present at about 2 dpf in the hybrid. In contrast, the pigmented cells appeared much later (5-6 dpf) in *A. australis *(Figure 5a, b).

Video recordings were made of several larvae, to observe the heart beat. The heart is bent in an S-shape, contracting regularly at a frequency of about 54 beats per minute in a 5 dpf larva (Additional file 1). There were no significant differences between the heart beat rates of 2 dpf and 5 dpf larvae, suggesting that the rate is based on the innate rhythm of heart muscle tissue.

### DNA analysis

**Figure 6:**
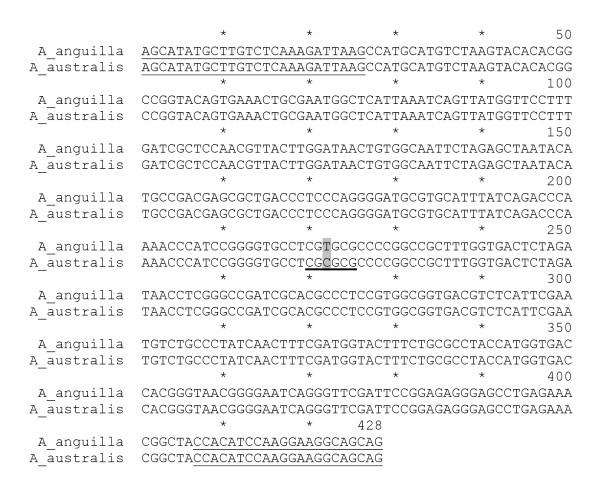
**PCR fragments from the 18 S rDNA genes**. Alignment of the PCR fragments amplified from the 18 S rDNA genes of *A. anguilla *and *A. australis *(location of forward and reverse PCR primers is single underlined; the restriction site for *BssH*II (position 220-225) is thick underlined; species-specific nucleotide difference at position 222 is highlighted in grey).

**Figure 7:**
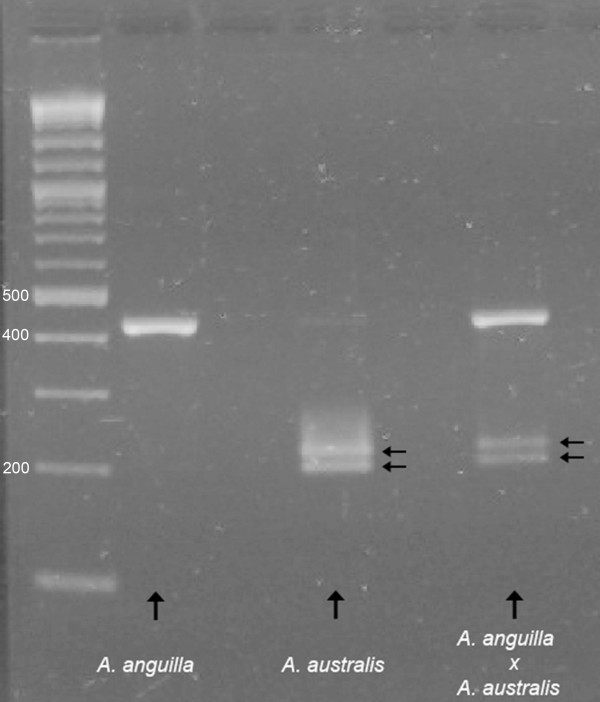
**PCR runs showing species-specific differences**. Identification of both parental species, *A. anguilla *and *A. australis*, and their hybrid, *A. anguilla × A. australis*, based on the species-specific nucleotide difference in nuclear 18 S rDNA. Arrows indicate the two fragments after restriction with the enzyme *BssH*II; the first lane indicates the position of the DNA size marker.

A species-specific nucleotide difference in the 18 S rDNA genes of *A. australis *and *A. anguilla *(Figure 6) allowed us to provide genetic evidence that we had produced hybrid offspring from the two species. The PCR product amplified from the 18 S rDNA gene is 428 bp long and has a single mismatch between *A. anguilla *and *A. australis *at position 222, resulting in a *BssH*II restriction site, specific for the *A. australis *product (Figure 6). *BssH*II digestion of the PCR product from *A. australis *therefore results in 207 bp and 221 bp fragments, whereas the 428 bp PCR product from *A. anguilla *is not digested by *BssH*II. As the hybrid species must contain both the *A. anguilla *and the *A. australis *18 S rDNA genes, three fragments were expected. Figure 7 clearly shows that the parental species *A. anguilla *and *A. australis*, as well as their hybrid, can be identified using the 18 S rDNA gene.

## Discussion

In this study, we succeeded in breeding *Anguilla australis *under artificial conditions and produced free swimming larvae of this species. In addition, hybrids of *A. australis *x *A. anguilla *were produced for the first time and genetic methods were used to confirm the existence of this hybrid. The hybrid larvae were kept alive for a maximum of 7 dpf and the larvae of *A. australis *for a maximum of 8 dpf.

Larvae of *A. japonica *reach the feeding stage at approximately 7 days after hatching [3]. This development is strongly temperature dependent and for *A. japonica *the optimal temperature was shown to be about 25°C [22-24]. It was noted that hatching also occurred at about 25°C [25] at the spawning site, suggesting that this may be the optimal temperature for early development. It was, however, noted that within a few days the larvae were distributed over an extremely large area at much lower temperatures [25], suggesting that early larvae are able to develop at a wide range of temperatures.

In our study, the larvae raised at 25°C (Figure 4c) were sufficiently developed to start feeding. In contrast, the 7 dpf larvae that were raised at 21°C had not yet reached the feeding stage and the head was still in a tilted position. The ten dpf larvae of *A. australis *-- collected by T. Kurwie at (illustrated in [26]) a prevailing temperature of 21°C -- were slightly more developed, although the 4^th ^ventricle was still large and the eyes were not as fully pigmented, as in the 8 dpf larvae reared at 25°C (Figure 4d). This clearly indicated that the main differences occur in the development of the head. Compared to 8 dpf larvae reared at 25°C, the mouths of 10 dpf larvae reared at 21°C were not fully developed, the teeth were just starting to form, and the mouth angle was not fully protruded anteriorly. This shows that development is highly temperature dependent and that larval development cannot be indicated by age alone.

Compared to other species, such as zebrafish [27] and medaka [28], development of the *Anguilla *head seems relatively slow. On the other hand, the appearance of the lateral neuromast cells, which are part of the mechanosensory system, was already observed on the flanks at approximately 1 dpf. After hatching, the larvae were very sensitive to vibrations, indicating that mechanoreception is well developed at this stage. Similar results were also reported for *A. australis *[10] and *A. japonica*. [29].

There were no visual differences in development and appearance between the two species other than the difference in timing of tail pigmentation, which occurred a few days earlier in the hybrid species than in *A. australis*. This seems to be a trait of the European eel, as a similar appearance of tail pigmentation was observed in *A. anguilla *[A Palstra, unpublished data] (Figure 5c). The reason for tail pigmentation occurring at this early stage remains unknown.

In studies on the natural hybrid of *A. anguilla *with *A. rostrata *[19,30], hybridization was validated by an independent method. This was not the case for recent artificial *Anguilla *hybridizations performed by Okamura et al. [18] and Lokman and Young [10]. Our results (Figure 7), based on the species-specific nucleotide difference in nuclear 18 S rDNA, show that both *A. anguilla *and *A. australis *and their hybrid *A. anguilla × A. australis *can been identified according to the method of Frankowski & Bastrop [30], in which the following fragments were produced: a single 18 S rDNA fragment for *A. anguilla*, two fragments for *A. australis*, and three fragments for the hybrid species, indicating that two alleles from both parent species were present in the hybrid.

There are still problems with artificial reproduction and larval rearing of Anguilla species, partly due to high individual variability in response to hormone treatments, and partly due to selecting the correct feed for larvae. Tanaka et al. [11] developed a reasonably successful feed for Japanese eel larvae although almost all larvae died before, or shortly after, the first feeding stage, which may have been due to the unnatural feeding methods that were employed. Possibly due to negative phototaxis, the larvae swim downwards towards the shark egg paste, where they encounter the food [[11]; Yoshimatsu, personal communication].

It is assumed that the natural food sources for leptocephalus larvae of *Anguilla spp*. are the oikopleura larvacean shelters (or 'marine snow'), which have been found in larval digestive tracts of several *Anguilloid *species [31]. Recent analysis of A. *anguilla *larval gut contents indicated that the diet of the smallest larvae consisted of a variety of plankton organisms, with Hydrozoa and Polycystinea species occurring most frequently [32]. So it seems that eel larvae may take a variety of available food from their immediate environment.

## Conclusions

The applied reproduction method resulted in healthy embryos and larvae of New Zealand short finned eels (*A. australis*) as well as hybrids from male European eels (*A. anguilla*) crossed with female New Zealand short finned eels. The developmental stages of eel embryos and larvae are described by means of high resolution images. In this paper we described the first production of hybrid larvae from male *A. anguilla *and female *A. australis *and their survival for up to 7 days post fertilization (dpf). Evidence for hybridization is based on a single nucleotide difference in the 18 S rDNA gene of both species. This is the first time that an artificial *Anguilla *hybrid has been validated by means of a genetic tool. Future work with this hybrid may provide further understanding of the reproductive mechanisms that affect breeding of the European eel, which is now on the CITES list of critically endangered species.

## Methods

### Eel collection

Silver females (n = 14; 80.1 ± 0.4 cm; 978.1 ± 19.5 g) and males (n = 8; 45.6 ± 1.4 cm; 172.5 ± 14.9 g) of New Zealand short-finned eels (*A. australis*) were caught in Lake Ellesmere in Christchurch, New Zealand, and transported to The Netherlands in aired plastic bags with a small amount of water, fitted into polystyrene boxes. Silver male European eels (*A. anguilla*) were purchased from the eel farm Royaal BV (Helmond, The Netherlands).

*Anguilla australis *females and males were kept together in a 1000 L tank filled with natural seawater, collected from Lake Grevelingen (30 ppt), and coupled to a 1500 L recirculation system (salinity 30 ppt, 21°C). The daily cycle was set with blue light (Philips special TLD Blue 36W/18) at 16:8 L:D. To compensate for the 11 h time difference between The Netherlands and New Zealand, the daily cycle was changed stepwise (1 h per week) to Central European Time (CET). *Anguilla anguilla *males (n = 15; 40.4 ± 0.6 cm; 118.8 ± 4.9 g) were kept in a 1500 L tank connected to a 2400 L recirculation system, in natural seawater (30 ppt, 21°C), under a complete dark regime. PVC pipes were introduced into both systems to provide refuges for animals. All animals were starved throughout the experiment, and treated on a weekly basis with Melafix (API aquarium pharmaceuticals, MARS Fishcare North America Inc., Chalfont, PA, USA) against infections. Prior to treatment, eels were anesthetized with 1-2 mL/L 10% clove oil (oil mixed 1:10 with absolute ethanol). At the start of this study all eels were tagged with passive transponders with unique identification numbers (Trovan, EID Aalten BV, Aalten, The Netherlands).

This experiment was approved by the animal ethical commission of the Leiden University (DEC# 08112).

### Hormone treatments

Female eels were distributed into four groups, with the starting point of hormonal treatment for each group being shifted one week forward, on a weekly basis. Treatment followed a modified version of Ohta's protocol [4,5]. On the first day of the week, females were weighed and injected intramuscularly (IM), at a point approximately 1 cm below the rostral attachment of the dorsal fin, with 20 mg salmon pituitary extract (SPE; Argent Labs, Redmond, WA, USA) per kg dissolved in 0.9% saline. When a 5% increase in body weight (BW) -- with respect to initial BW -- was reached, females were transferred to a separate 400 L tank, connected to the same system. The BW was measured the day after the transfer and/or 2 days later. When a 10% increase in BW was reached, an oocyte sample was collected by means of inserting a cannula (polyethylene tube, inner diameter 1.4 mm) through the oviduct. Oocytes were checked under a microscope to ascertain developmental stages. When migration of the germinal vesicle -- still with many oil droplets in the oocytes (stage 3/4 in European eel according to [9]) -- was observed, the female was primed with a single injection of SPE (20 mg/kg dissolved in 1 mL 0.9% saline). After 24 h, ovulation was induced by means of intraperitoneal injections of 17, 20β-dihydroxy-4-pregnen-3-one (DHP 2 mg/kg, Sigma-Aldrich BV, Zwijndrecht, The Netherlands) dissolved in DMSO, administered at six to eight locations.

Male New-Zealand short-finned eels were treated according to a modified version of Ohta's protocol [4,5]. They were subjected to a weekly IM injection procedure (at a site approximately 0.5-1 cm below the rostral attachment of the dorsal fin) with 250 IU human chorionic gonadotropin (hCG, Sigma-Aldrich BV, Zwijndrecht, The Netherlands) dissolved in 0.1 mL 0.9% saline. Males were injected every week for up to 9 weeks and checked for spermiation by hand stripping. After 9 weeks, all males produced milt and injections were stopped until a female was ready to spawn. (This reduced handling stress; it was also noted that the sperm quality did not decline much during the "holding" period). On the day before the eggs were to be stripped, two to three males that demonstrated high sperm motility were selected and stimulated with a single booster dose of 500 IU dissolved in 0.1 mL saline. Sperm motility was determined visually under a microscope, after mixing a drop of sperm with a drop of seawater. Only sperm with at least 50% motility (continuous activity of > 50% of spermatozoa) was used for fertilization.

For the production of a hybrid between *A. australis *and *A. anguilla*, 15 farmed male European eels received weekly intraperitoneal (IP) hCG injections, according to the protocol for European eel [9], at a dose of 200 IU/male, followed by a booster dose of 1,000 IU hCG (in 0.2 ml 0.9% saline) 24 h before a fertilization trial.

### Artificial fertilization and larval rearing

Two to three males per species were hand stripped 24 h after the hCG booster injection. Milt was collected in a syringe (10 mL) and kept on ice or in the refrigerator for a maximum of 48 h. Sperm motility was checked prior to fertilization by means of microscopic examination. Females were expected to ovulate between 11 and 15 h after the final injection with DHP. The artificial fertilization programme was terminated in cases when female spawning only commenced after more than 18 h after the final injection, which is indicative of low fertility and hatchability [5]. After the final injection, females were checked hourly for egg release, by gently pressing on the abdomen near the vent. When a female showed an ovarian plug, the plug was gently removed. Eggs were collected in plastic, pre-weighed, sterilized bowls. The first flow of eggs (~50 g) was not used for fertilization. The combined egg weight was determined after all eggs had been stripped.

The collected sperm was added to dry eggs in bowls and mixed. Fresh seawater (35 ppt, 20°C) was added, and after approximately 3-4 min the eggs were transferred into buckets with fresh (sterile) seawater (~20 L). A net (of mesh size 600 μm) was used to separate floating eggs from sinking eggs. The former were transferred, after 30-45 min, to another bucket containing fresh seawater. Finally, the eggs were transferred to 1 L glass beakers and/or 200 mL Petri dishes, for observation. At this stage they were kept in complete darkness at 21°C and a salinity of 35 ppt. Approximately 24 hours after incubation, the water was refreshed by transferring the still-floating eggs into new glass beakers or Petri dishes. During the trial, all white or sunken eggs were removed. A portion of eggs from one batch were also reared at 25°C.

### DNA analysis

As described by Frankowski & Bastrop [30], parental species and their hybrids can be identified by means of polymorphism in nuclear 18 S rDNA. For our study we used a slightly different protocol, as described below.

Total DNA was isolated and purified from ten whole hybrid larvae and fin-clips of parental specimens, using a DNeasy Blood & Tissue Kit (Qiagen). The polymerase chain reaction (PCR) was performed using the FastStart High Fidelity PCR System protocol (Roche) and an amplification profile consisting of denaturation for 3 min at 94°C, 35 cycles of 30 s at 94°C, 30 s at 60°C and 1 min at 72°C, followed by 5 min at 72°C for final extension. Amplification was carried out according to the manufacturer's instructions in 50 μL 1 × FastStart High Fidelity Reaction Buffer containing 1.8 mM MgCl_2_, 2.5 U FastStart High Fidelity Enzyme Blend, 1 μg chromosomal DNA, 0.4 μM of the 18 S rDNA forward and reverse primers, and 0.2 mM dNTPs.

The sense (5'-AGC ATA TGC TTG TCT CAA AGA TTA AG-3') and antisense (5'-CTG CTG CCT TCC TTG GAT GTG G-3") primers were based on NCBI accession numbers FM946133 (*A. australis*) and FM946070 (*A. anguilla*) [23]. The PCR product was purified using a QIAquick PCR Purification Kit (Qiagen) and 0.5 μg of the purified fragments were digested with two units of the restricting enzyme *BssH*II (New England Biolabs Inc) according to the manufacturer's instructions. Restriction enzyme digestion was conducted in 10 μL reaction buffer for 1 h at 37°C. DNA fragments were made visible using a 2% agarose gel.

## Authors' contributions

EB is a PhD student working on early development of *Anguilla *species. He carried out reproduction experiments, DNA-assays, developmental analyses and writing of the manuscript. SB is a biologist, who carried out reproduction experiments (with EB). TK is senior scientist at MTI; she designed the reproduction experiments. RD is a senior scientist at ZF; he designed the PCR-probe and assay and corrected the manuscript. PD is director of MTI; he planned transport of NZ eels and was involved in the reproduction design. At the time when these experiments were undertaken AP was a postdoctoral student involved in the reproduction design. HP is head of the Molecular Cell Biology Section and was involved in producing high resolution pictures and videos of embryos and larvae. GvdT is associate professor, supervisor of EB, and project leader. All authors read and approved the final manuscript.

## Supplementary Material

Additional file 1 **Video clip of a 5 dpf embryo of *A. australis *with a heart rate of 54 beats per minute**. The heart is S-shaped and beats regularly. Click here for file
